# Meanings of recovery and post-traumatic growth in people with lived experience of eating disorders: a qualitative study

**DOI:** 10.1186/s40337-025-01258-2

**Published:** 2025-04-23

**Authors:** Chiara Tosi, Davide Patanè, Ludovica Natali, Valentina Meregalli, Valentina Cardi

**Affiliations:** 1 Department of General Psychology, University of Padova, Padova, Italy; 2 Centre for Research in Eating and Weight Disorders, Department of Psychological Medicine Institute of Psychiatry, Psychology & Neuroscience, King’s College London, London, UK

**Keywords:** Anorexia nervosa, Bulimia nervosa, Binge eating disorder, Interviews, Thematic analysis.

## Abstract

**Background:**

Conceptualizations of recovery from an Eating Disorder (ED) have focused traditionally on symptom alleviation and restoration of physical health. In recent years, through patient involvement, this definition has been broadened to incorporate changes in psychological processes and overall wellbeing. This study used a qualitative approach to explore the meanings of recovery among people with lived experience of an ED. Areas of personal growth after the illness were explored, with reference to the theoretical framework of Post-Traumatic Growth (PTG).

**Methods:**

Nineteen participants (mean age 24.37; range: 18–50), who self-identified as recovered from an ED (mean time since recovery = 33.68 months, standard deviation = 31.67 months; range = 5-130 months), completed a demographic questionnaire and were asked to discuss their experiences of recovery and areas of PTG in a structured interview. Reflexive Thematic Analysis (TA) was used to identify common themes and sub themes.

**Results:**

Four overarching themes and 14 sub themes were identified. Recovery was defined as a non-linear process marked by changes in eating behaviours (e.g., greater flexibility), and more effective abilities to manage painful thoughts and emotions. Elements of PTG were included in the experience of recovery, particularly through a reconstituted, authentic and meaningful sense of self following the ED.

**Conclusions:**

People with lived experience of an ED provided a broad definition of recovery, which encompassed symptom restoration, as well as significant changes in psychological processes and elements of PTG. These findings have the potential to inform the development or refinement of recovery-focused treatments.

**Supplementary Information:**

The online version contains supplementary material available at 10.1186/s40337-025-01258-2.

## Abstract (plain language summary)

Eating disorders (EDs) are complex conditions impacting both mental and physical health. Recovery from an ED extends beyond overcoming eating-related issues; it involves personal growth and discovering new life meanings. In this study, 19 individuals who identified themselves as recovered from an ED were interviewed to explore their experience of recovery and areas of personal growth after the illness. Participants described recovery as a challenging yet rewarding journey, marked by setbacks, breakthroughs, and a shift in self-perception. They reported developing a more flexible and positive relationship with food, reducing the influence of negative thoughts on their self-evaluation, and finding healthier ways to manage emotions. Support from therapists and loved ones were crucial in fostering confidence, change and self-awareness. Many participants became more resilient and connected with others and expressed a renewed appreciation for life and hope for the future. These findings highlight that recovery from an ED involves more than symptom resolution and physical health restoration; it is about a profound sense of personal transformation. This understanding can enhance the acceptability, uptake and breadth of current psychological treatments for people with EDs.

**Supplementary Information:**

The online version contains supplementary material available at 10.1186/s40337-025-01258-2.

## Background

Eating disorders (EDs) are complex psychiatric conditions which posit a heavy burden on patients and their families. According to the latest report of the Global Burden of Disease study, despite treatment advances, there has been an increase in years of life lost (YLDs) and mortality rates for these conditions compared to other mental health disorders, where these indices have remained stable [1]. A possible reason why even evidence-based treatments work sub-optimally is because treatment goals might not take fully into account patients’ preferences and values. In recent years, for example, qualitative studies on patients’ narratives have made it possible to broaden the definition of recovery beyond improvements in core eating disorder symptoms, i.e., flexible eating and weight restoration, to include psychosocial outcomes, such as enhanced self-esteem, emotional and interpersonal skills, and strengthened social relationships and future outlooks [2–6]. Clinicians and researchers too, have started embracing a definition of recovery as an evolving process, rather than a binary state (reached/not reached), in which psychological changes play a critical role [3, 7–9]. However, due to a longstanding tradition of measuring recovery through medical parameters and binomial outcomes (i.e., recovered vs. not recovered), the eating disorder literature still lacks an established framework to map out the various facets of the recovery process.

A known model to define the positive changes that an individual can experience after a traumatic event, such as an illness, is the post-traumatic growth (PTG) model [10, 11]. Post-traumatic growth is defined as the “positive psychological change experienced as a result of the struggle with highly challenging life circumstances” [11]. Based on this definition, a traumatic event can trigger personal positive changes in the way in which an individual sees the self, the future and others, leading to transformations compared to the pre-trauma identity [12–14]. The model predicts that PTG derives from the person’s efforts to incorporate the trauma into the personal life narrative. In part, this process is enabled by conscious rumination, i.e., a focused, intentional reflection on trauma-related themes which aligns with personally meaningful goals and recur independently of external triggers [15]. Studies on PTG have also found associations between PTG and certain personality traits, such as extraversion and openness; greater abilities to cope with distress and negative emotions; and social support, particularly when stable and enduring [15].

PTG has been documented in response to physical illnesses, such as oncological [16] and infectious diseases [17], natural disasters [18], abuse or interpersonal violence [19, 20], and described using five dimensions, i.e., improved relationships with others, new possibilities for life, a stronger sense of personal resilience, spiritual growth, and enhanced appreciation of life. Only very recently, this model has been extended to mental health recovery [21], enabling researchers to identify further dimensions, such as self-discovery, integration of illness experiences into one’s identity, and increased engagement and commitment to self-management of wellbeing.

The use of this framework to expand the understanding of recovery from an eating disorder seems particularly appropriate, considering that the disruptions caused by the eating disorder on the individual and their families can resemble those caused by traumatic events. Also, recovery from an eating disorder must include a process of psychological change which leads to a novel and more authentic sense of self, in similar ways to the personal growth which might follow a traumatic experience, such as a physical illness. However, to date, only one study has explored PTG following an eating disorder, combining quantitative and qualitative data and conceptualizing eating disorders as resulting from traumatic events [22]. The results of the study documented the presence of PTG, with no significant differences between *recovered people* or *people in recovery*. In the study, the most endorsed dimension of PTG was of interpersonal nature and related to a sense of belonging and meaningful relationships. As expected, a correlation was found between PTG scores and the use of purposeful and deliberate rumination [22].

The current study contributed to the literature on PTG in eating disorders, by employing a semi-structured interview to explore people’s experience of recovery from an eating disorder and their sense of personal growth during and following recovery.

## Methods

### Study design

This study used a qualitative design. A semi-structured interview was developed by the study team with the goal of exploring participants’ views of recovery and post-traumatic growth. Answers to the interview questions were video-recorded and transcribed. Transcripts were analysed using Reflexive Thematic Analysis (TA) to systematically identify, categorise, and interpret patterns of meaning (themes) across the dataset [23].

### Participants

This study is part of a larger project investigating PTG in individuals with eating disorders, which used both qualitative methods and structured validated questionnaires. Participants were recruited from the general population through social media (e.g., Instagram, Facebook, WhatsApp, Threads, and X). Inclusion criteria were (a) age 18 or older, (b) a self-reported lifetime diagnosis of an eating disorder according to the Diagnostic and Statistical Manual of Mental Disorders, Fifth Edition (DSM-5), (c) having received treatment for the eating disorder, and (d) a self-reported status of recovery from the eating disorder. Thirty-four individuals expressed interest in taking part. Interviews were conducted until thematic saturation (i.e. the point at which no new themes or codes emerge from data [23], resulting in a total of 19 participants. Ethical approval was obtained from the Psychology Research Ethics Committee of the University of Padova, in Italy (approval number: 298-b). The study adhered to the Declaration of Helsinki and GDPR (EU, 2016/679) standards. Written consent was obtained from all participants, who were informed of their right to withdraw at any time from the study and reminded of their option to skip questions during the interviews.

### Data collection

Participants completed an online questionnaire on Qualtrics XM platform and provided social-demographic information about age and sex, as well as basic clinical data to ensure eligibility. They were asked about their diagnosis, the duration of the eating disorder, whether they considered themselves recovered, the type and number of treatment cycles they received, and the factors they viewed as most influential to their recovery. Qualitative data were subsequently gathered through a semi-structured interview conducted online with each participant, via a video call, between February and July 2024. Each individual interview lasted approximately 50 min. Interviews began with a few ice-breaking questions exploring the experience of living with an eating disorder, to then progress to examine personal definitions of recovery and dimensions of post-traumatic growth experienced during and following recovery. A full outline of the interview is provided as Supplementary material (see Supplementary Material 1). All interviews were recorded and transcribed verbatim for analysis. Transcripts were pseudo-anonymised and potentially identifying information, including names and specific locations were removed.

### Data analysis

The descriptive characteristics of the sample (*n* = 19) were analysed using JASP (version 0.18.3). Means and standard deviations (SD) were calculated for variables such as age, illness duration, time since recovery, and cycles of treatment received, while frequency was used for variables including biological sex and type of treatment received.

### Thematic analysis (TA)

A hybrid approach to thematic analysis (TA) was employed, combining both deductive and inductive components. Based on the conceptual framework of PTG proposed by Tedeschi and Calhoun (1996), a deductive approach was first applied. At the same time, participants’ narratives were used to guide the exploration inductively, revealing new themes. Through this dual approach, both latent and semantic themes were identified [23]. The six-phase process recommended by Braun and Clarke [24] was used. Following transcription and familiarization with the data, each interview was coded by two independent raters (C.T. and D.P.). Each rater reviewed and coded key segments individually. The raters then met to compare perspectives and collaboratively establish a coding framework. The full dataset underwent two cycles of coding, after which codes were grouped into preliminary themes and sub-themes. The thematic structure was refined iteratively, with regular sessions to review and adjust the coding framework, ensuring coherence between raters. In the final phases, raters organized the refined codes into final themes and sub-themes. There were no significant disagreements, and themes were largely consistent between raters.

### Reflexivity

Research team members recognize that their own perspectives and experiences could have influenced data analysis and synthesis. Both coders (C.T. and D.P.) have clinical and research experience in the field of eating disorders, which informed their understanding of participants’ narratives but also called for careful reflections to mitigate potential bias. A reflexive approach was used, with team members openly discussing thoughts and reactions throughout the research process during regular meetings. The 15-point checklist by Braun and Clarke [24] was used to ensure rigor and quality when conducting thematic analysis. Additionally, to avoid potential interpretation bias from the literature, the coders consulted PTG materials after the first round of coding and initial discussions. This study adopted an abductive approach within an interpretivist epistemological framework, and it integrated a naturalistic interpretation with theory-informed analysis. Grounded in the assumption that meaning is constructed through participants’ narratives, the study inductively explored recovery and post-traumatic growth while applying a theoretical framework to expand understanding. This approach enabled an iterative process of engagement with empirical data and the existing theory, ensuring a nuanced interpretation with no causal inferences.

## Results

Nineteen participants (18 females and 1 male; mean age 24.37, range: 18–50) who self-identified as recovered from an eating disorder (Mean time since recovery: 33.68 months, SD = 31.67, range = 5-130 months) and who had received treatment for the illness in the past, prior to recovery, completed the demographic questionnaire. Five people reported multiple lifetime diagnoses of eating disorders. The most often reported diagnosis was anorexia nervosa (reported by 14 people), followed by binge eating disorder (five people), bulimia nervosa (three people), avoidant/restrictive food intake disorder (two people) and eating disorder not otherwise specified (two people). Most (*n* = 14, 73.69%) underwent multiple treatments: 11 people reported having received outpatient treatment, five people daycare, nine people intensive care (i.e., hospitalization), 16 people psychotherapy and 10 people nutritional support (Supplementary Materials 2 and 3).

**Fig. 1:**
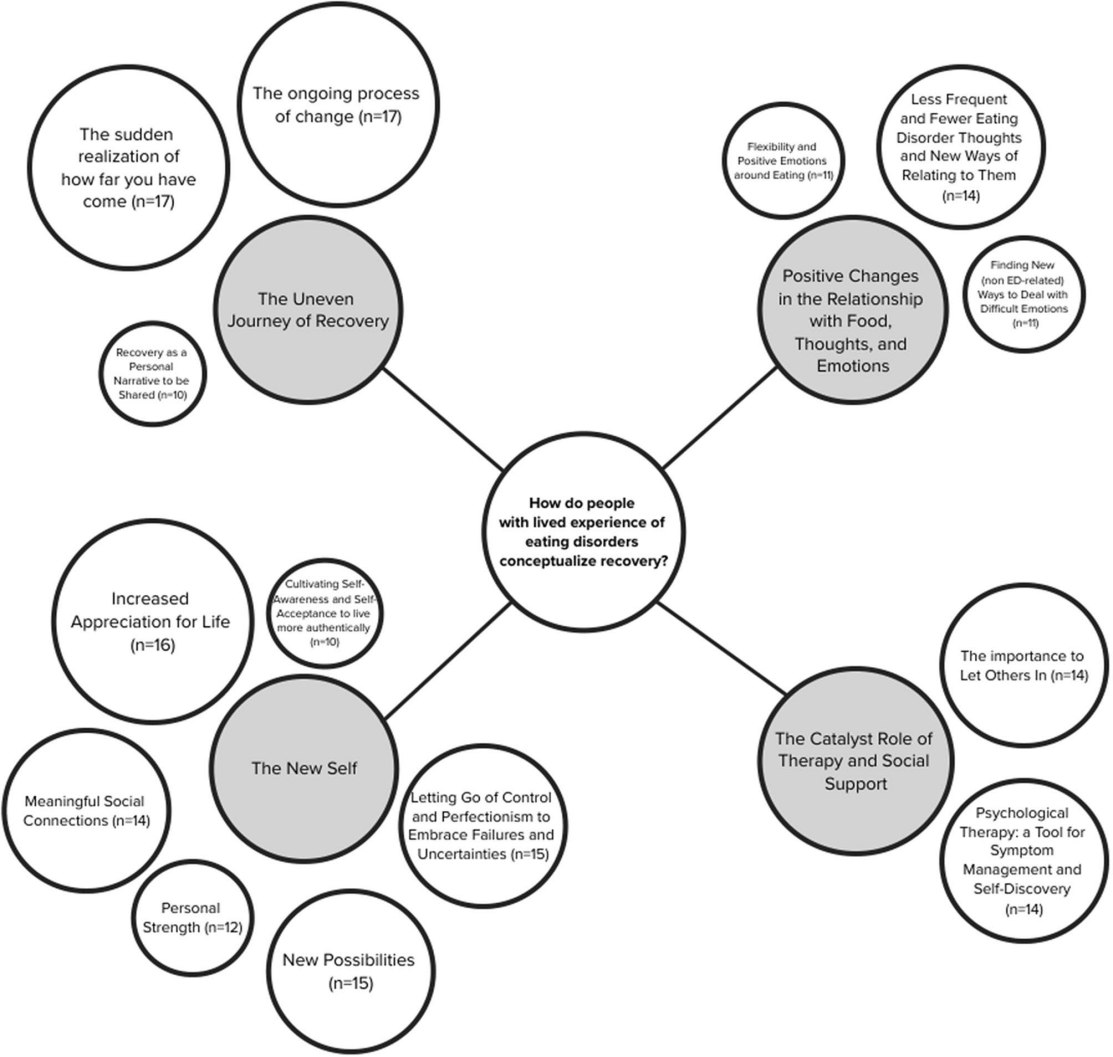
Map of the themes, sub themes and related frequencies. *Notes.* Grey circles: themes; White circles: sub themes; Circles’ measure corresponds to citation frequency: bigger circles mean higher number of citations

Four overarching themes and 14 sub themes were identified (Fig. 1) through qualitative analysis.

Participants generally defined recovery as a non-linear process which had led to positive changes in the frequency of, and the relationship with, food and eating disorder-related thoughts. Recovery was framed as an empowering process characterised by the enhanced ability to recognize and navigate through triggering situations successfully. Elements of PTG were included in the experience of recovery, particularly through a reconstituted new sense of self following the eating disorder. Representative quotes for each theme and sub-themes are presented below, with participant names replaced by coded identifiers (see Table 1 for further details).

**Table 1:** Themes, subthemes, frequencies and representative quotes

Theme	Subtheme	Frequency (%)	Quote(s)
THE UNEVEN JOURNEY OF RECOVERY	1a. The ongoing process of change	17 (89.47%)	“It was slow, but I don’t mean in terms of years, I mean in terms of the overall process. It was a slow thing because you’d think, “Okay, I feel better,” but then the thoughts come back, and you start again. So, in my opinion, the process is truly slow” (part. 4) “There were always periods when things were a bit better and periods when they were worse, but over time, it gradually stabilized” (part. 6) “It’s a process where you almost don’t realize, as you work on yourself, that you might be starting to detach. Yet, at the same time, this thing still keeps you somewhat tied to it” (part. 15)
	1b. The sudden realisation of how far you have come	17 (89.47%)	“When I started feeling good and right with myself—in the sense that I no longer cared about judgments, what people said, the “buts” or the “ifs”—because I was who I was, and I am who I am” (part. 1) “There was a very beautiful and special moment, about two years ago, when one day I looked in the mirror and felt nothing. I wasn’t searching for flaws or problems anymore” (part. 8) “They took my measurements, weighed me, and literally, when they said, “Your blood pressure is good, your weight is good,” I gave the same importance to my blood pressure as to my weight. It was like I had forgotten about it, and that’s what matters” (part. 11)
	1c. Recovery as a personal narrative to be shared	10 (52.63%)	“Then I started looking at them and thought, “No, wait, I want everyone to see this, I want them to know how much I struggled.” But since the struggle isn’t something you show, it’s something you share, I wanted them to see this. So that desire to share that I had healed really filled me up” (part. 5) “I’m aware that I’m the same as yesterday, and tomorrow I’ll be the same as today. Nothing changes” (part. 8) “By giving a voice to these things, because every time I have the chance, I participate. I’m glad that, compared to a few years ago, I did an interview—not one like this, but still about eating disorders” (part. 15)
POSITIVE CHANGES IN THE RELATIONSHIP WITH FOOD, THOUGHTS AND EMOTIONS	2a. Flexibility and positive emotions around eating	11 (57.89%)	“The journey that a person often goes through in their approach to food and the meaning it takes on, because, of course, for me, the meaning has changed” (part. 2) “Accepting the ability to allow yourself something that makes you feel powerful by depriving yourself of it— for me, that was a turning point” (part. 7) “There was a precise moment when I changed the need… the craving for bingeing into “I just want to eat something in front of a movie,” and that was the key moment for me” (part. 15)
	2b. Less frequent and fewer eating-disorder thoughts and new ways of relating to them	14 (73.68%)	“In the moment when your voice comes back, you think, “Wow, but in my life, I have everything: I do the things I love, I have people who care about me, I’m at peace. Why should I ruin it?” (part. 4) “I think the important thing is not giving it the same weight you did before and knowing that there’s more to life than how we appear aesthetically” (part. 6) “I realized that food, which once seemed enormous, was becoming smaller and smaller every day” (part. 10)
	2c. Finding new (non-eating disorder-related) ways to deal with difficult emotions	11 (57.89%)	“When something happens, it’s the most critical moment when one can revert to using certain strategies learned when they were younger. It helps me a lot, maybe I do it without even realizing, like listening to things that were said to me often” (part. 2) “I’ve learned to manage those ups and downs that appeared both in life and in the illness—meaning in the healing process—differently” (part. 7) “Now, when things happen that make me angry, or when I overreact, I always try to pause for a moment […]. It’s about accepting the crappy moments and embracing the very beautiful ones” (part. 8)
THE CATALYST ROLE OF THERAPY AND SOCIAL SUPPORT	3a. The importance to let others in	14 (73.68%)	“You can’t do it alone; that’s something you have to get out of your head because, unfortunately, it’s not something possible. You need the right support” (part. 3) “Family helped, friends who were there for me […]. It’s about fully trusting the people around you who truly want to help you heal” (part. 6) “The help of others, of loved ones, whether family, friends, or acquaintances, I think that in various contexts, if you’re lucky, you can find warmth, which is important to get through difficult moments” (part. 16)
	3b. Psychological therapy: a tool for symptom management and self-discovery	14 (73.68%)	“Definitely therapy […]. But we worked on me […]. It’s really about healing the deep wounds” (part. 8) “The psychotherapy journey, in particular, because it helped me find the source, the initial reason. […] We started to also modify the coping mechanisms for my problems” (part. 15) “The first moment I felt free to let out everything I had inside without fear, despite the questions I was asked or the process of delving into particularly painful aspects, which wasn’t always pleasant—quite the opposite—I realized that being asked uncomfortable questions is part of the process. And what happens afterward is gaining much greater self-awareness” (part. 16)
THE NEW SELF	4a. Increased appreciation for life	16 (84.21%)	“I took the wheel of my life because I only have this one” (part. 1). “Discovering that there’s life beyond the disorder” (part. 6). “After going through that difficult period, I realized that, yes, I needed to be seen and everything, but it wasn’t that I really wanted to die. That was definitely a confirmation” (part. 17).
	4b. Meaningful social connections	14 (73.68%)	“I’m more aware in my relationships with others, which have obviously become more genuine” (part. 2) “It’s improved, but not in the sense that we’re more attached; it’s a bit more balanced, we’ve found a healthy relationship” (part. 6) “So, definitely, the relationships I have now are helping me a lot. It’s an experience that has allowed me to find deeper relationships and connections” (part. 7)
	4c. Personal strength	12 (63.16%)	“Definitely, I’m more confident in myself now” (part. 8) “It’s something that strengthens you a lot. For me, the fact that I noticed it on my own, that I went to therapy and came out of it, gives me incredible strength” (part. 10) “If I set my mind to it, I can even help myself. I can do that kind of work” (part. 17)
	4d. New possibilities	15 (78.95%)	“Before, during the period of illness, I had no future perspective. Everything was very focused on the present or small future actions, so it was a projection that could only go to the next day. Now, future perspectives have opened up in many ways” (part. 3) “I realized I had healed when I started making plans for the future, saying, “Okay, tomorrow, okay, in a week, but where will I be in a year?” I began to put things in perspective—reaching tomorrow, starting to hope again, looking ahead” (part. 11) “During the period of the eating disorder, I couldn’t see a future, so I would’ve been dead then, I couldn’t see any future at all. But now I have a clear goal in mind, I know what I want to do, and I see a future ahead of me” (part. 13)
	4e. Letting go of control and perfectionism to embrace failures and uncertainties	15 (78,95%)	“Another thing that weighed heavily on me was accepting the fact that there was no longer that part of me that controlled things” (part. 4) “You make mistakes, it’s important to accept the mistake, which doesn’t mean you’ve thrown everything away. On the contrary, you analyze the mistake, see what happened, the causes of the mistake, and move forward. These mistakes will become fewer and fewer. You really learn” (part. 10) “Not having control over things… it was definitely something that, at the time, I didn’t like, but it helped me. I still put myself in the mindset that I don’t have to be able to do everything, to handle it, to patch it up. It’s okay to feel bad sometimes; it’s okay to need help” (part. 17)
	4 f. Cultivating self-awareness and self-acceptance to live more authentically	10 (52.63%)	“But little by little, you accept yourself, you say, “This is how I am, what can I do?” (part. 4) “It allowed me to heal the wounds little by little; to accept the things I don’t like, learn to embrace them, and change how I relate to those things” (part. 8) “I like myself, I’m happy with who I am, and I think that everything I’ve gone through, the difficulties, the pain, has led me to become the person I am now. I also appreciate those parts of me that once may have been seen as flaws, but now I know they are very important aspects I’m proud of” (part. 16)

Note. Frequency: number of participants who mentioned the theme/19

### Theme 1: the uneven journey of recovery

This theme captures participants’ perceptions of recovery, defined as a non-linear process, marked by relapses and ambivalence towards change. Participants described elements of their treatment journey, which often involved multiple attempts and different approaches. They highlighted the ongoing nature of recovery while recognizing achieving specific milestones along the way. They described their recovery as an integrated and coherent story which could inspire others to overcome the illness.

#### The ongoing process of change

Participants described recovery as a gradual process, characterized by ups and downs and ambivalence, as quoted in the following sentence: *“There were a lot of relapses and ups and downs along the way. There were times when I was more determined and convinced*,* I was following the right direction*,* and other times when discouragement or fear took over”* (Participant 3). Through recovery, participants experienced difficulties in stepping away from the comfort zone of the disorder, and taking risks in uncertain, disorder-free, circumstances. Some highlighted the difficulty of drawing a clear line between illness and recovery which made it difficult to define themselves as “recovered”, e.g., *“It’s not really something where you just say*,* “Okay*,* I’m recovered.” It comes gradually*,* as you start reclaiming all the things you couldn’t do before.”* (Participant 9). Moreover, they recognized the ongoing nature of the recovery process, which continued beyond the remission of disordered eating behaviours, e.g., *“Certainly*,* with many setbacks along the way*,* moments of highs and lows*,* of course*,* periods when I was more inclined and convinced that I was moving in the right direction*,* and other moments when discouragement prevailed or when fear of moving forward took over*,* as well as a bit of lack of motivation. It’s not linear; it continues not to be linear. It’s somewhat cyclical*,* but one thing is certain: I will never go back to where I started many years ago. That*,* for sure*,* will not happen”* (Participant 3).

#### The sudden realisation of how Far you have come

Participants identified insightful moments during their recovery journey, when they suddenly realised how far they had come. These moments provided them with feelings of reassurance, hope and motivation to continue challenging the eating disorder. They sometimes noticed unexpected changes in food-related behaviours, e.g.,: *“A particular moment was when I returned home after being hospitalised. It was in the simplest things*,* like tasting a bit of sauce from the lid of a tomato jar with my finger. My dad told me “What are you doing?” He was surprised by it*,* but it was indeed something so simple. However*,* considering how I used to be*,* tasting something with one finger… if I wasn’t asked to I would never have done it!”* (Participant 3). Others mentioned the new positive feelings related to the experience of eating, e.g., *“There has been a specific moment where I changed my need of… I switched from binge eating craving to simply “eating something while watching a movie”*,* that has been a key moment for me”* (Participant 15). Some referred to their ability to cope with difficult situations using other strategies than resorting to food (“*I realized that I was*,* let’s say*,* healed when there were some difficulties in my family*,* and my attitude changed”* (Participant 6), and feeling that the illness no longer defined them, e.g., “*I understood that life*,* a life consumed only by illness*,* no longer belonged to me. That was the moment when I perhaps realized that… I was on the path to healing*” (Participant 19).

#### Recovery as a personal narrative to be shared

Both the eating disorder and the recovery process were described by participants as an integrated narrative defining a new sense of self, shaped both by struggles and growth experienced throughout recovery. They were understood as life chapters to share with others, e.g., *“In my opinion*,* showing people who suffer from these disorders that there are others who share the same mechanisms is important because it makes you feel less alone […] Talking about food*,* giving advice to others… it makes me feel good*,* and I really enjoy it. You know it was a part of you*,* a part of your life. You don’t deny it at all; on the contrary*,* that’s exactly where you start again”* (Participant 10). By re-framing the eating disorder as a traumatic event which contributed to personal growth, participants were able to reconcile with their past and incorporate the illness into their current personal identity: *“I think things happened a certain way*,* but now I’m here*,* and I’m here with an awareness and a desire to live*,* to enjoy everything that’s to come—something I didn’t even have before the illness”* (Participant 16). Many identified self-disclosure as a pivotal moment in the recovery process, serving a dual purpose. Sharing the journey to recovery not only contributed to personal healing but also enabled them to foster connection and offer support to others, e.g., *“It’s important for someone who has suffered to have the strength and courage to speak about it*,* as this can inspire and support those who are struggling to overcome their challenges. In my opinion*,* this is crucial because by sharing our experiences we can make a meaningful difference in helping others”* (Participant 9).

### Theme 2: positive changes in the relationship with food, thoughts and emotions

This theme reflects the positive changes participants experienced in their relationship with food, thoughts, and emotions during recovery. Many noted a shift towards greater flexibility in their approach to eating, which was perceived as a more enjoyable activity. They also reported less frequent and fewer negative and intrusive thoughts related to food and body image and the development of healthier coping strategies for managing stress and negative emotions.

#### Flexibility and positive emotions around eating

Participants mentioned increased flexibility around food, with eating becoming more spontaneous and less subject to rigid rules or prohibitions, e.g., *“I’m no longer so fixated on the portion size or type of food I’m having during a meal*,* which used to be such a big deal for me before”* (Participant 3), and also a pleasurable experience “*I started eating not just to survive*,* but for the pleasure of eating*” (Participant 14), or *“Sometimes I eat a little more because I enjoy eating*” (Participant 10). They also reported feelings of empowerment from the ability to manage eating behaviours, e.g., “*Developing routines and incorporating meals into those routines to manage them effectively*” (Participant 18), “*I’m happy anyway*,* and I know it’s not the ice cream eating me*,* but me eating the ice cream*,* which is different*” (Participant 1).

#### Less frequent and fewer eating-disorder thoughts and new ways of relating to them

Most participants mentioned positive changes in relation to the eating-disorder thoughts. Some described less frequent, fewer, when not a proper disappearance of these negative thoughts, e.g., “*It’s as if my recovery journey has helped me silence those voices”* (Participant 1), or “*It is now years since I became normal again*,* that I don’t even have thoughts*,* I mean food-related thoughts*,* I just don’t have them anymore*” (Participant 2). Others described acquiring new tools to manage these thoughts during stressful periods, e.g., *“It’s a bit like an Achille’s heel*,* that is normally silenced but appears during stressful moments*,* even unconsciously*,* as disturbing thoughts. I think that the moment you learn to manage them it is when you realise you are truly recovered and will no longer need them”* (Participant 2)”. An example of such tools is the ability to engage in a dialogue with the thoughts rather than avoiding or suppressing them, e.g., “*I’m able to have a back-and-forth with the thoughts that might cross my mind*,* and this helps me*” (Participant 5), or “*I would answer myself when I had intrusive thoughts […] and eventually*,* after fighting it for a while*,* it stopped*” (Participant 17). Many reported no longer believing in these thoughts or managing them with greater assertiveness: “*There are moments of difficulty when they come up and… um… but what changes is*,* first of all*,* no longer believing in it… I mean*,* believing that I am worth something based on my weight*,* that I can’t be loved unless I am at my ideal weight or a certain size*” (Participant 18).

#### Finding new (non-eating disorder-related) ways to deal with difficult emotions

Participants highlighted the importance of developing positive emotion regulation skills as part of the recovery process. The recognition of how difficult emotional states could trigger dangerous eating behaviours was described as key to develop novel strategies for effective emotional regulation and resilience: *“Regulating emotions is important. Everything that goes from identifying to managing triggers. You need to gain control over these feelings”* (Participant 17). The positive strategies included seeking help when needed, managing emotional ups and downs, and learning to tolerate/experience unpleasant feelings while also embracing both good and bad moments: *“I stop and ask myself what’s wrong*,* whether there’s something deeper*,* an unmet need or if there’s an emotion I haven’t been able to express”* (Participant 18).

### Theme 3: the catalyst role of therapy and social support

This theme highlights the pivotal role of therapy and the support of significant others, such as family members and partners, in the recovery process. Participants emphasized the importance of trusting and relying on professionals and their loved ones, particularly during the illness.

#### The importance to let others in

Participants emphasized the importance of being able to trust and rely on others, including therapists, healthcare professionals, and family members, to support their recovery, particularly when their awareness of the illness was limited in the acute phase of the disorder, e.g., *“It’s not easy*,* and there will be many relapses*,* but keeping trust in those who care for you that understand what’s best for you the only way is to get through it. In those moments*,* you can’t rely on yourself because your thoughts are fading and not real. To get “the real” in your mind*,* you need to trust someone else”* (Participant 2). Important barriers to trusting others included fear of misunderstanding and negative judgment, e.g., “*Listen to those around you*,* it’s not all criticism*,* what they want for you isn’t bad*” (Participant 13), or “*Realize*,* above all*,* that the people who want to help you are not here to judge you. I’ve seen so many things that I’ve seen it all*,* but they are human beings here to help other human beings. So*,* no one is here to judge you specifically; you’re just another patient*” (Participant 11).

#### Psychological therapy: a tool for symptom management and self-discovery

Psychological therapy was described as a safe space to explore both eating disorder symptoms and underlying issues, including painful past experiences, emotional struggles, and negative thought patterns. Psychological therapy was regarded as crucial to develop helpful tools to navigate life challenges in healthier ways, e.g., *“I’ve really felt the effectiveness of psychotherapy. It’s been a great help. I’ve probably found the right therapist for me; therapy has given me tools to deal with anything that comes in my way” (Participant 7)*​​. Moreover, it offered the opportunity to explore different facets of the self and foster emotional growth, self-discovery and self-acceptance, e.g., *“I have been in psychotherapy for a long time*,* which was the most crucial part for me because it allowed me to address many aspects of myself despite being only 15 at the time. It helped me build the life I have now”* (Participant 2).

### Theme 4: the new self

Despite acknowledging the traumatic nature of the illness, participants recognized the role of the eating disorder in shaping their current sense of self and identity. They described positive psychological changes following recovery, which touched upon several areas of their lives. In terms of PTG domains, firstly described by Tedeschi and Calhoun (1996) all were identified as outcomes of the recovery process, except for the spiritual dimension, which was mentioned only by one participant. Many described gaining greater authenticity, flexibility, self-awareness and self-acceptance through the illness and the recovery process.

#### Increased appreciation for life

Participants described a renewed attachment to life and a desire to embrace it fully following recovery, e.g., *“thinking that life is so important and that it doesn’t matter what you have to go through*,* you can make it. Life is always worth living*,* it’s an enormous gift”* (Participant 8), “*Now I’m truly hungry for life*,* for experiences*,* to take back everything that the illness took from me*,* including enthusiasm*” (Participant 16). Many spoke of finding joy in small, everyday moments, e.g., *“I definitely appreciate a lot of small things. Even when something negative happens*,* I always try to stay optimistic and focus on getting the best of the situations”* (Participant 16), “*So*,* this experience has definitely taught me to appreciate life and the small moments*” (Participant 9), and starting to feel at ease in different situations: *“I have truly found peace in my life*,* in the things I do in my life*,* so work*,* I feel at peace*,* I have my friends*,* I do the things I enjoy […] In my opinion*,* the important thing is to find in your own life the things that bring you peace”* (Participant 4).

#### Meaningful social connections

Participants described many positive changes in their relationships with others, which became more meaningful, authentic, and enjoyable during and after recovery. They learned to prioritize relationships that foster mutual support, empathy, and trust, e.g., *“It gave me confidence in my relationships with close friends and the fact that they were interested and wanted to help me. With my family*,* thanks to psychotherapy*,* I was able to express some needs I had*,* which actually led to an improvement”* (Participant 17). Many talked about feeling more secure and confident in their ability to connect to others and recognised the opportunity to benefit from social support, with no fear of judgment. As one participant reflected, “*I realised that I needed to build strong relationships and create meaningful connections to others*,* including those I had left behind. I understood that if I didn’t allow others into my life*,* I would never achieve what I truly needed: an authentic bond with people*” (Participant 16).

#### Personal strength

Through recovery and the opportunity to overcome challenges, participants developed a stronger sense of self-efficacy, e.g., *“It helped me rediscover my willpower*,* which has always been my trait: when I set my mind to something*,* I commit and succeed. My dedication is constant and precise*,* and I invest a great deal into what I do. Although it was previously invested in a wrong way*,* if applied correctly*,* it could truly bear fruit”* (Participant 3), and the hope to successfully navigate obstacles in life: “*I say*,* ‘Wow*,* I’ve overcome an illness*,* so I can do this too.’ So*,* I still see myself as strong*,* capable*,* and especially determined to achieve the goals I set for myself*” (Participant 9).

#### New possibilities

The recovery process enabled participants to envision the future as a realm of possibilities, allowing them to make plans, set goals, and define objectives, e.g.,*“When I was ill*,* there was no future*,* only the present. But now I have goals that give me a perspective on the future”* (Participant 19). They recognized the limitations imposed on them by the eating disorder, e.g.,*“Now I can build something for the future*,* which*,* of course*,* you can’t do with an eating disorder”* (Participant 9) and developed a clearer understanding of their own will, needs, and values “*Today*,* on the other hand*,* I think about my future*,* I want to think about it*,* I imagine it—it’s something completely different”* (Participant 4), including the motivation to sustain recovery in the future, e.g., *“Planning a bit for the future was kind of the drive for survival”* (Participant 14).

#### Letting go of control and perfectionism to embrace failures and uncertainties

The recovery process fostered greater cognitive flexibility, tolerance of uncertainty, and less need of control among participants, e.g., *“With joy*,* I can say that I’ve fully embraced life again*,* without needing to have control over everything*,* welcoming the unexpected and all that comes with it”* (Participant 16). This represented a significant shift from the rigid patterns of thoughts and behaviours that had previously dominated their lives, where control and predictability served as key coping mechanisms to deal with painful and difficult emotions. Participants described developing the ability to accept situations as they are and posing less emphasis on the need to strive for perfection or exert control over every detail, e.g., *“I think a big part of it was learning to accept uncertainty and ambiguity*,* letting go of the need to control everything. It also allowed me to listen to myself*,* something I don’t think I had ever truly done before.”* (Participant 2). Additionally, they adopted a more flexible attitude toward mistakes, perceiving them as opportunities for growth rather than failures, cultivating a more balanced and adaptive approach to life.

#### Cultivating self-awareness and self-acceptance to live more authentically

Participants highlighted the profound impact of recovery in fostering self- acceptance and validation of one’s own emotions and needs. As stated by one participant: “the fact of saying " I need to be *name*, to be seen as *name*,* authentic*,* in all my weaknesses”* (Participant 16). They reported learning to listen to their desires, accept and prioritize their needs, and act according to their authentic selves. This involved acknowledging their value independently of other’s judgement, e.g., *“It’s me living my life*,* and that’s okay”* (Participant 10). This transformation allowed participants to approach life with integrity and a greater sense of emotional attunement, e.g., *“This experience has helped me connect with my needs and listen to myself”* (Participant 7). By fostering self-compassion and authenticity, the recovery journey provided the foundation for a more meaningful and self-affirming way of living, e.g., *“I believe that if I hadn’t experienced everything I went through*,* I probably wouldn’t have reached the awareness I have now about what I want and about myself”* (Participant 16).

## Discussion

The aim of this study was to understand recovery and areas of post-traumatic growth in individuals with lived experience of eating disorders. Participants described recovery as a non-linear and ongoing journey, where setbacks and progress coexisted [25–28]. They endorsed a broad model of recovery, encompassing remission of eating disorder symptoms as well as increased flexibility, emotion regulation and changes in the relationship with the “eating disorder voice [29, 30]. They described changes in managing their thoughts (from avoidance to assertiveness) and painful emotions, and moments of sudden realization of their flexibility around eating. The acknowledgement of these “turning points” and the ability to notice positive changes have been found to exert a positive impact on motivation to sustain recovery over time [31, 32].

In addition to these changes, participants identified domains of PTG as key to the emergence of a new sense of self, following recovery. Recovery fostered a renewed attachment to life, helped finding joy in small moments, and provided a sense of peace and fulfilment. It allowed participants to envision a purposeful future, promoting sustained personal growth and enhanced quality of life. Social connections expanded, grounded in trust and mutual support, while personal strength reflected in willpower and self-confidence. Cognitive flexibility and self-acceptance emerged as key processes to sustain growth and adapt to different life domains, providing a foundation for navigating challenges and complexity. These changes have been documented in the eating disorder literature, especially in qualitative studies of patients’ personal accounts [3, 4, 22, 33–35]. Psychological therapy was recognized as essential in participants’ recovery, offering tools to manage eating disorder symptoms and to address underlying suffering. It was described as a safe space for self-reflection and a pathway to self-acceptance, crucial for fostering growth and healing. Therapy likely facilitated intentional rumination, which is known to promote post-traumatic growth [11]. Participants also emphasized the importance of relying on a supportive network during recovery, including family, friends, and peers, which provided emotional and practical support. This aligns with existing findings on the critical role of support networks in the recovery process [7, 36]. Self-disclosure also emerged as a key milestone in recovery. It enabled individuals to recognize small positive steps and integrate the experience of illness and recovery into one re-defined identity. Many participants valued sharing their stories with others, also to instil hope and motivation to change in people currently suffering from an eating disorder [33, 37–43].

This study expands the existing literature on post-traumatic growth in the context of physical and mental illness. Despite the paucity of studies in the field of mental health, findings in eating disorders align with what has been found in other severe psychiatric disorders, such as psychosis (e.g [21], where post-traumatic growth is mostly driven by interpersonal factors [44]). Similarly, in women with a history of addiction and victimization, the experience of post-traumatic growth seems prevalent and associated with a sense of community and lower levels of psychological distress [45]. However, the lack of studies in other psychiatric conditions limit the conclusions that can be drawn on the predictors, correlates and outcomes of post-traumatic growth as a transdiagnostic factor. Qualitative studies have been mostly employed and generated interesting hypotheses. Longitudinal and mechanistic investigations are needed to examine the temporal and casual associations between illness, recovery and positive psychological changes following recovery.

### Limitations

Eligibility to take part in this study was based on participants’ self-reported characteristics, i.e., a past diagnosis of an eating disorder and having received treatment for the eating disorder. No clinical records were required to corroborate patients’ reports because the focus of interest was on personal narratives. Broad inclusion criteria were adopted, with no restrictions based on age, duration of illness and recovery. This is because no a priory hypotheses had been generated with regards to how post-traumatic growth might be affected by these variables. Based on findings, themes and subthemes were not characterised by differences in these characteristics. This is suggestive of the idea that post-traumatic growth is affected by psychological variables, some of which might be unrelated to the core eating disorders psychopathology (e.g., deliberate rumination). Comorbidities were not investigated and therefore it cannot be concluded how these impacted on process of change.

Despite the use of broad and inclusive participation criteria, recruitment might have been biased by people’s high levels of motivation to participate and awareness of the illness. Similarly, raters’ personal and professional characteristics might have biased the interpretation of findings. Furthermore, the interview was based on a specific framework (i.e., the post-traumatic growth framework) which might have limited the investigation of other factors contributing to recovery.

Despite these limitations, the study provides meaningful insights into the experience of recovery and psychological growth following an eating disorder, with the potential of informing recovery-based interventions to instil hope and provide models of change.

## Conclusions

The findings of this study emphasize the need to incorporate psychological dimensions of change into the definition of recovery from an eating disorder. The PTG approach provided a suitable framework to expand the understanding of recovery, beyond symptom remission and participants’ global functioning. Recovery emerged as a transformative process that integrates the experience of the illness into one’s life narrative, fostering a new, meaningful and authentic sense of self. Psychological therapy resulted crucial in guiding patients through the definition of their own identity, while addressing disorder-specific symptoms. Adopting a positive, hopeful approach to recovery - focused on metacognitive processing and identity discovery - may offer significant benefits to currently ill people.

## Electronic supplementary material

Below is the link to the electronic supplementary material.

Supplementary Material 1

## Data Availability

The datasets used and/or analysed during the current study are available from the corresponding author on reasonable request.

## Abbreviations

PTG: Post-Traumatic Growth
EDs: Eating Disorders
TA: Reflexive Thematic Analysis
DSM-5: Diagnostic and Statistical Manual of Mental Disorders, Fifth Edition
