# Supplementary material for: Meanings of recovery and post-traumatic growth in people with lived experience of eating disorders: a qualitative study
Source: J Eat Disord. 2025 Apr 23;13:70. doi: 10.1186/s40337-025-01258-2 (PMC12016052; doi:10.1186/s40337-025-01258-2)
Supplement: Supplementary file 1 — Supplementary Material 1 [file 40337_2025_1258_MOESM1_ESM.docx]

**Supplementary material 1**. Integral version of the semi-structured interview.

Good morning, thank you for agreeing to participate in this interview. This is a structured interview, meaning I will ask you a series of pre-determined questions with the goal of understanding your healing experience and how your life has changed after the eating disorder. As indicated in the informed consent text, if there are any questions you do not wish to answer or if you feel uncomfortable at any point, you may stop your participation at any time.

1. I would like to start by asking you what it was like for you to suffer from an eating disorder (e.g., what do you remember most vividly about that experience?).

Thank you very much for sharing these memories/thoughts. Now, I would like to ask you to think about your healing process.

1. When did you realize that you had recovered from the eating disorder? (e.g., what made you think/feel that way? Do you remember a specific moment when you realized you were healed?).
2. How did your recovery happen? What kind of journey was it (e.g., a quick or slow process, linear or winding)?
3. What kind of relationship do you think exists between the illness and recovery? For example, would you describe the illness and recovery as two distinct processes or as ones that overlap? In your experience, were you able to clearly distinguish the boundaries between the two?
4. What was the most challenging part of the recovery process?
5. What do you think contributed the most to your recovery?
6. What helps you maintain your state of recovery day by day?
7. How has your life changed after the illness? For example, how has your relationship with significant others, such as your loved ones, changed?
8. How has your outlook on life and the future changed after the illness?
9. How has your self-image changed after the illness?
10. Is there something in particular you would like to say to someone who is currently struggling with an eating disorder and is afraid they won’t be able to make it (for example, afraid of facing the fear that the disorder will leave an insurmountable void or that it will never truly go away)?
11. Is there anything else you would like to add before we say goodbye?

We sincerely thank you for the time you have dedicated to us, and we hope that you liked the interview.

**Supplementary material 2.** Types of diagnosis (frequencies)

|  | F (%) |
| --- | --- |
| AN | 14 (73.69%) |
| BN | 3 (15.79%) |
| BED | 5 (26.32%) |
| ARFID | 2 (10.53%) |
| EDNOS | 1 (5.26%) |
| Unknown Diagnosis | 1 (5.26%) |

*Note: AN: Anorexia Nervosa; BN: Bulimia Nervosa; BED: Binge Eating Disorder; ARFID: Avoidant/restrictive food intake disorder; EDNOS: Eating Disorder Not Otherwise Specified.*

**Supplementary Material 3.** Types of treatments (frequencies)

|  | F (%) |
| --- | --- |
| Outpatient treatment | 11 (57.89%) |
| Day hospital Treatment | 5 (26.32%) |
| Intensive treatment (hospitalization) | 9 (47.37%) |
| Psychotherapy | 16 (84.21%) |
| Nutritional support | 10 (52.63%) |
